# Neuroinflammation in fungal infections: from pathogen recognition to pathological manifestations

**DOI:** 10.1242/dmm.052344

**Published:** 2025-10-09

**Authors:** Rachael Dangarembizi, Amalia Awala, Anja de Lange

**Affiliations:** ^1^ Division of Physiological Sciences, Department of Human Biology, Faculty of Health Sciences, University of Cape Town, Cape Town 7125, South Africa; ^2^ Neuroscience Institute, Faculty of Health Sciences, University of Cape Town, Cape Town 7125, South Africa; ^3^ CMM AFRICA Medical Mycology Research Unit, Institute of Infectious Diseases and Molecular Medicine, Faculty of Health Sciences, University of Cape Town, Cape Town 7125, South Africa

**Keywords:** CNS mycoses, Neuroinflammation, Antifungal immunity, Neuroimmune, Neuroinfections

## Abstract

Fungal diseases of the central nervous system (CNS) are associated with severe neurological damage and death in immunocompromised hosts, yet they remain neglected in research and policy. Neuroinflammation, a common clinical feature of fungal infection, has been implicated as a key driver of brain injury, but the mechanisms underlying its contribution to pathology are not well understood. The aim of this Review is to discuss the double-edged role of neuroinflammation in the pathogenesis of fungal infections. We provide an overview of the immune barriers that protect the CNS from fungal infection, the fungal strategies that enable immune evasion and neuroinvasion, and the complex mechanisms underlying the development of neuroinflammation during fungal infection. Finally, we explore how both insufficient and excessive neuroinflammatory responses drive neuropathology, and we conclude by outlining current challenges as well as potential directions for advancing future research in this overlooked field.

## Teaser

**Summary:** Fungal infections of the central nervous system are under-recognised drivers of neurological disease and mortality. This Review highlights how neuroinflammation, an essential host response for antifungal defence, also contributes to neuropathology.

## Introduction

Invasive fungal infections are a leading cause of death in immunosuppressed individuals (Brown et al., 2024). An estimated 6.55 million people are affected by life-threatening fungal diseases, resulting in an estimated 3.8 million deaths (Denning, 2024). Although these infections account for less than 20% of all invasive fungal cases (Sharma, 2010), central nervous system (CNS) infections are often associated with fatal outcomes (Nathan et al., 2021; Sharma, 2010). Symptomatic CNS infections that are caused by fungi are linked to a disproportionately greater risk of morbidity and mortality than bacterial, viral and parasitic CNS infections (Sharma, 2010). Several factors have driven the increase in CNS infections of a fungal origin over the past 50 years: (1) a significant increase in the population affected by immunosuppressive conditions, such as human immunodeficiency virus (HIV)/acquired immunodeficiency syndrome (AIDS) (Zhao et al., 2023); (2) advancements in medical technologies and procedures that heighten the risk of fungal infection, including invasive brain surgeries and prolonged use of immunosuppressive therapies (Lionakis and Levitz, 2018); and (3) the coronavirus disease 2019 (COVID-19) pandemic, as severe acute respiratory syndrome coronavirus 2 (SARS-CoV-2) infections have been associated with increased susceptibility to fungal infections of the CNS (for a comprehensive review on this topic, see Baddley et al., 2021). These challenges are further exacerbated by the emergence of new fungal species that infect the CNS, which is driven by climate change and the growing problem of antifungal resistance among established pathogens (Lionakis and Levitz, 2018).

Although fungi are found everywhere, there is regional variability in the burden of fungal disease that is driven by various factors, including fungal ecology and distribution, socioeconomic factors affecting access to health care and timely diagnosis, and HIV prevalence (Denning, 2024). In 2022 the World Health Organization (WHO) compiled the ‘WHO Fungal Priority Pathogens List’ (FPPL), a list of 19 fungal pathogens that pose as major health threats to the global population but have been neglected in research and policy (World Health Organization, 2022). Remarkably, all the pathogens listed in the WHO-FPPL have been reported to infect the human CNS (see Table 1), although most fungal infections of the human CNS are caused by *Cryptococcus* spp., *Candida* spp., *Aspergillus* spp., *Histoplasma* spp. and *Coccidioides* spp. (Rajasingham et al., 2017; Pappas et al., 2016; Charalambous et al., 2018).

**Table 1.** Central nervous system involvement among fungal pathogens listed in the World Health Organization's Fungal Priority Pathogens List

WHO-FPPL classification	Fungal pathogen	CNS infection	References
Critical priority	*Cryptococcus neoformans*	Yes	Charalambous et al., 2018; Dao et al., 2024
	*Aspergillus fumigatus*	Yes	Khandwala et al., 2024; Meena et al., 2021
	*Candida auris*	Yes	Lino et al., 2024; Mirhendi et al., 2022
	*Candida albicans*	Yes	Charalambous et al., 2018; Roilides et al., 2019
High priority	*Nakaseomyces glabratus* (*formerly Candida glabrata*)	Yes	Maziero et al., 2025
	Eumycetoma causative agents	Yes (including *Aspergillus fumigatus*, *Scedosporium* spp. and *Fusarium* spp., among others)	Hoenigl et al., 2023; Jenks et al., 2018; Khandwala et al., 2024; Lino et al., 2024; Meena et al., 2021; van de Sande, 2013
	*Fusarium* spp.	Yes	Hoenigl et al., 2023; Jenks et al., 2018; Khandwala et al., 2024
	*Candida parapsilosis*	Yes	Roilides et al., 2019
	*Histoplasma* spp.	Yes	Charalambous et al., 2018
	*Mucorales*	Yes	Khandwala et al., 2024
	*Candida tropicalis*	Yes	Roilides et al., 2019
Medium priority	*Scedosporium* spp.	Yes	Gao et al., 2022; Khandwala et al., 2024; Lino et al., 2024
	*Lomentospora prolificans*	Yes	Lino et al., 2024
	*Coccidioides* spp.	Yes	Charalambous et al., 2018; Sivasubramanian et al., 2023
	*Candida krusei*	Yes	Pelletier et al., 2005
	*Cryptococcus gattii*	Yes	Dao et al., 2024
	*Talaromyces marneffei*	Yes	Li et al., 2020; Lino et al., 2024
	*Pneumocystis jirovecii*	Yes	Nasnas et al., 2012; Vidal et al., 2000
	*Paracoccidioides brasiliensis*	Yes	de Almeida et al., 2017

CNS, central nervous system; WHO-FPPL, World Health Organization's Fungal Priority Pathogens List.

CNS fungal infections, like many other CNS infections, typically originate in peripheral infection sites – such as the lungs, skin, intestinal tract or vaginal mucosa – and later disseminate to the brain haematogenously (Nathan et al., 2021). In some patients, direct contiguous spread to the CNS can occur from adjacent anatomical sites such as the paranasal sinuses, orbits, petromastoid region and retropharyngeal space (Chimelli and Mahler-Araújo, 1997; Dotis and Roilides, 2007). Additionally, direct inoculation of the CNS may arise during traumatic events, neurosurgical procedures or intensive care interventions (Chimelli and Mahler-Araújo, 1997). Fungal infections of the CNS are particularly difficult to treat, largely owing to the lack of antifungal agents with reliable penetration into the cerebrospinal fluid (CSF) or brain parenchyma, coupled with pharmacokinetic variability, resistance issues and host toxicity (Ashley, 2019).

CNS fungal infections (also known as CNS mycoses) are therefore often associated with severe neurological damage that leads to death or debilitating dysfunction in those who survive (Sharma, 2010). Clinical syndromes associated with fungal infections of the CNS vary depending on the fungal pathogen, but the most common syndromes include basal meningitis, hydrocephalus, parenchymal abscesses and granulomas, and stroke syndromes and myelopathies (Chimelli and Mahler-Araújo, 1997; Dotis and Roilides, 2007). The immunopathological mechanisms that contribute to CNS injury during fungal infections remain poorly understood, mainly owing to the limited availability of human data. However, emerging evidence indicates that neuroinflammation, a common clinical manifestation during fungal infections, is a potential driver of CNS injury in fungal disease (Dotis and Roilides, 2007; Reyes and Shinohara, 2022).

This Review aims to explore the interactions between fungal pathogens and the CNS, with a specific focus on the double-edged role of neuroinflammation in the pathogenesis of CNS fungal infection. We provide an updated overview of current knowledge on CNS protection against fungal invasion, strategies employed by fungi to overcome these defences, and the brain's innate ability to combat fungal infection and persistence. Additionally, we examine how improperly regulated neuroinflammatory responses can contribute to neurological damage. This Review is particularly timely given the rising global incidence of invasive fungal infections, including those affecting the CNS, which remain under-recognised and difficult to treat, highlighting an urgent clinical need to consolidate current knowledge and explore novel therapeutic strategies.

## CNS immunity against fungal pathogens

**Fig. 1.**
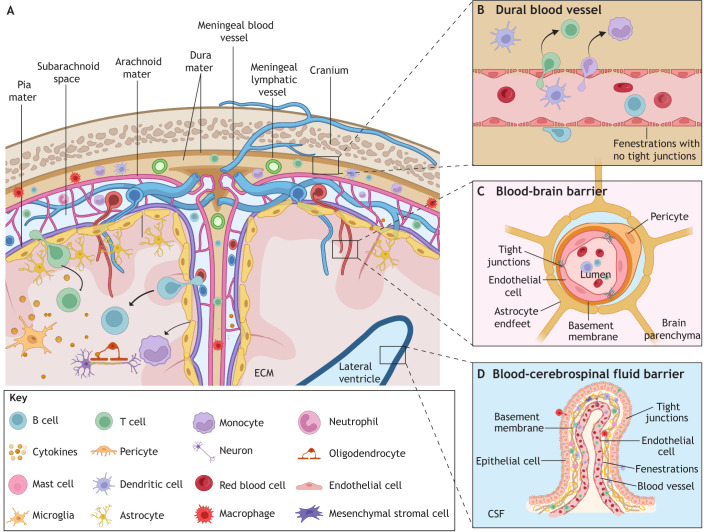
**Central nervous system (CNS) immunity against fungal pathogens.** (A) The three layers of meninges (dura mater, arachnoid mater and pia mater) restrict fungal entry into the parenchyma while permitting immune cells (resident and immune cells recruited from the circulating blood) to conduct immune surveillance. (B) The fenestrated meningeal blood vessels, which allow immune cells to access the meningeal compartment, might also act as a gateway for fungal infection. (C) The blood–brain barrier is localised to small vessels that supply the parenchyma, and it is formed primarily by tight junctions between endothelial cells, with contribution from pericytes, astrocyte end-feet and basement membranes. (D) The blood–cerebrospinal fluid barrier, which lines the choroid plexus in the ventricles, is formed by the tight junctions of cuboidal epithelial cells and fenestrated endothelium. CNS immune barriers serve the primary function of restricting fungal entry into the CNS, but some fungal pathogens have developed strategies to breach these barriers and cross into the parenchyma. In the brain parenchyma are recognised and destroyed by the coordinated efforts of resident and recruited immune cells. CSF, cerebrospinal fluid; ECM, extracellular matrix. Created in BioRender by Dangarembizi, R. (2025). https://BioRender.com/625jztn. This figure was sublicensed under CC-BY 4.0 terms.

The CNS has historically been regarded as an immune-privileged site, based on early experimental findings showing that autologous or xenogeneic grafts were better tolerated in the brain than in peripheral tissues. In 1921, Shirai observed that rat sarcoma cells survived when implanted into the CNS parenchyma, but were rejected when transplanted into peripheral sites such as skin and muscle (Shirai, 1921). Building on this, Medawar later demonstrated in a rabbit model that allogeneic grafts placed in the brain elicited significantly less immune rejection than those placed peripherally. However, he also showed that intracerebral grafts were rapidly rejected in animals that had been immunologically primed, revealing that the CNS is not immunologically inert but rather sequestered from immune surveillance under normal conditions (Medawar, 1948). The concept of immune privilege was further reinforced by three other lines of evidence: the presence of a robust physical barrier, the blood–brain barrier (BBB), which separated the CNS from systemic circulation (Ehrlich, 1885; Goldmann, 1913; Reese and Karnovsky, 1967); the presumed lack of resident immune cells capable of antigen presentation within the parenchyma (Ransohoff and Engelhardt, 2012); and the initial belief that the brain lacked a lymphatic drainage system (for a comprehensive review on the topic of immune privilege, see Louveau et al., 2015a). However, more than a century of research has since demonstrated that CNS immune privilege reflects not an absence of immune responses but a highly organised and tightly regulated system that aims to protect neural tissue while minimising collateral damage. CNS neuroimmune function involves the coordinated and elaborately controlled action of barriers, fluids, resident and recruited immune cells, and the extracellular matrix (ECM), all of which collaboratively maintain CNS homeostasis and defend it against pathogens. As we next discuss, the immune responses of the CNS to fungal pathogens rely on multiple layers of protection: the meninges, which cover the brain and spinal cord; the BBB and the blood–CSF barrier (BCSFB), both supported by the ECM; and resident and recruited immune cells (Fig. 1).

### Meningeal immunity

The meninges are composed of three membrane layers (the dura mater, arachnoid mater and pia mater) that protect the CNS (Fig. 1). However, they are no longer regarded as merely structural barriers but as immunologically active regions that are of crucial importance for pathogen recognition and clearance (Alves de Lima et al., 2020). The dura mater, the outermost meningeal layer, contains fenestrated blood vessels that allow immune cells to access the meningeal compartment (Fig. 1B). Recent single-cell RNA sequencing and flow cytometry studies have identified a diverse repertoire of immune cells in the murine (Van Hove et al., 2019) and human (Wang et al., 2022a) dura, including monocytes, CD4^+^ and CD8^+^ T cells, neutrophils, dendritic cells, border-associated macrophages (BAMs), natural killer cells and B cells (Van Hove et al., 2019). The dura mater also contains lymphatic vessels that drain CSF, macromolecules, microbes and immune cells to cervical lymph nodes, thereby facilitating immune communication with peripheral sites (Aspelund et al., 2015; Louveau et al., 2015b). Subdural meninges (arachnoid and pia mater) harbour a similar but less abundant population of immune cells, including monocytes, BAMs, dendritic cells, natural killer cells, T cells and B cells (Van Hove et al., 2019). These subdural meningeal layers have intercellular tight junctions that prevent free movement of molecules and immune cells, and also limit the spread of pathogens from the dura mater towards the brain parenchyma (Rua and McGavern, 2018). During infection, the relatively non-restrictive fenestrated dural vessels can act as a gateway for fungal pathogen entry into the meningeal compartment, triggering inflammation in the meninges (meningitis). For this reason, patients with *Cryptococcus*, *Candida* and *Coccidioides* infection may develop meningitis prior to, or even unaccompanied by, encephalitis (Sharma, 2010). The meningeal compartment functions as an immunological hub, playing a key role in pathogen sensing, antigen presentation, immune cell trafficking and activation, and cytokine production, topics that we discuss in more detail in later sections in this Review. The meninges also act as an early immunological barrier against fungal infections, facilitating fungal clearance and preventing the progression of fungal infections into the parenchyma, which is further protected by the BBB and BCSFB.

### The blood–brain barrier and blood–cerebrospinal fluid barrier

In the mammalian brain, pathogen entry into the parenchyma is restricted by the presence of the BBB on parenchymal capillary and post capillary vessels (Abbott, 2005). The BBB is an immunologically active barrier that is structurally comprised of endothelial cells, two layers of basement membranes, pericytes and astrocytic end feet (Daneman and Prat, 2015) (Fig. 1C). The functional integrity of the BBB is maintained by the presence of adherens junctions and tight junctions between endothelial cells, which impede the movement of bloodborne fungal pathogens (Daneman and Prat, 2015; Klein and Hunter, 2017) (Fig. 1C). Most regions in the brain have a full, intact BBB except for a few areas adjacent to the third and fourth ventricles that have fenestrated capillaries (Ufnal and Skrzypecki, 2014). These areas with a ‘leaky’ BBB are commonly referred to as circumventricular organs (CVOs), and they include the area postrema, pineal gland, median eminence, organ of the vascular lamina terminalis and subfornical organ (Ufnal and Skrzypecki, 2014). Fenestration ensures greater passive permeability, which facilitates blood solute sensing and the secretion of molecules (e.g. hormones), but fenestration also makes CVOs potential windows for circulating fungal pathogens to enter into the brain (Bentivoglio et al., 2018).

The BCSFB plays a crucial role in protecting the CNS by regulating the exchange of substances between the blood and CSF, thereby preventing pathogens and toxins from entering the CNS parenchyma. The BCSFB is primarily located at the choroid plexus and consists of two layers: a fenestrated, ‘leaky’ capillary endothelium, which allows the selective filtration of molecules necessary for CSF formation; and a layer of cuboidal epithelial cells with tight junctions that form the true barrier that regulates the movement of solutes, immune cells and pathogens (Fig. 1D) (Engelhardt and Coisne, 2011; Ghersi-Egea et al., 2018). These layers are supported by an intervening basement membrane, which provides structural integrity and additional filtration properties (Engelhardt and Coisne, 2011; Ghersi-Egea et al., 2018). The CSF compartment is an active immunological surveillance site, in which various peripheral immune cells – including T cells, dendritic cells and macrophages – are involved in pathogen detection (Thompson et al., 2022). CSF circulates in the ventricles and goes to the subarachnoid space, where it enters the meningeal and perivascular compartments to facilitate waste clearance and immune surveillance. The CSF is then drained via dural lymphatic vessels, which transport it to the cervical lymph nodes, linking CNS immunity with the peripheral immune system (Engelhardt et al., 2016).

Although it may be tempting to think of the BBB and the BCSFB as impenetrable fortified walls, some fungal pathogens can breach these barriers to cross into the parenchyma. First, the permeability of both barriers changes significantly during infection and inflammation (Varatharaj and Galea, 2017), and some fungal pathogens have devised mechanisms to cross over by altering the integrity of these barriers. We will take a closer look at the mechanisms used by fungal pathogens to cross into the brain in later sections of this Review.

### Neuroimmune cell populations

The immunity of the CNS to fungi involves both resident and recruited immune cells, which detect, respond to and clear fungal pathogens. All cells within the CNS – including glia, neurons and cells that make up the neurovascular system – have immune capabilities, but microglia, astrocytes and BAMs are the cells that mainly mediate innate inflammatory responses in the CNS (Wang et al., 2023; Zheng et al., 2022). Here, we outline the general roles of neuroimmune cells in combating infection, with a more detailed discussion of their contribution to inflammatory activation provided in later sections.

**Fig. 2.**
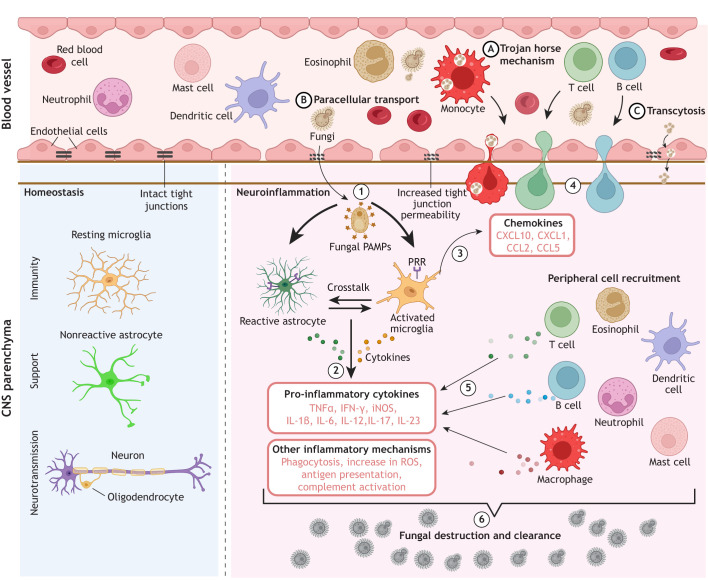
**Neuroinflammatory activation during fungal infection.** Under healthy homeostatic conditions (left panel), the blood–brain barrier has intact tight junctions that prevent pathogen entry and maintain normal immune homeostasis. Microglia provide immune surveillance, and astrocytes support homeostasis to ensure optimal neuronal function. Fungal infection of the CNS (right panel) can occur via several routes, which include a Trojan horse mechanism (A), paracellular transport (B) and transcytosis (C). (1) Fungal recognition of pathogen-associated molecular patterns (PAMPs) by pathogen recognition receptors (PRRs) on resident immune cells of the brain (e.g. microglia and astrocytes). Once activated, these cells release pro-inflammatory cytokines and chemokines. (2) Pro-inflammatory cytokines trigger phagocytosis, antigen presentation and complement activation; chemokines initiate (3) the recruitment and (4) migration of peripheral immune cells such as T cells, monocytes, neutrophils and eosinophils to the brain. (5) Recruited immune cells release pro-inflammatory cytokines, reactive oxygen species (ROS) and other chemicals to help clear the pathogen. (6) As a result, appropriately regulated neuroinflammatory processes help with destruction and clearance of fungal pathogens. CCL, C-C motif chemokine ligand; CXCL, C-X-C motif chemokine ligand; IFN-γ, interferon-gamma; IL, interleukin; iNOS, inducible nitric oxide synthase; TNF-α, tumour necrosis factor-alpha. Created in BioRender by Dangarembizi, R. (2025). https://BioRender.com/c55qy77. This figure was sublicensed under CC-BY 4.0 terms.

Microglia are the only resident myeloid cells in the parenchyma, and they are the first line of defence against invading fungal pathogens. Microglia originate from the embryonic yolk sac, have self-renewing capability, and perform vital homeostatic and phagocytic functions in the brain (Ajami et al., 2007; Klein et al., 2017). Similar to their mononuclear phagocytic counterparts from the peripheral immune system (monocytes, macrophages and dendritic cells), microglia recognise fungal pathogens through a repertoire of pathogen recognition receptors (PRRs), have antigen-presenting capabilities, and secrete cytokines and chemokines when activated (Koutsouras et al., 2017; Yamasaki et al., 2014) (Fig. 2).

The second major type of immunocompetent cells in the CNS is the astrocyte. Astrocytes were long considered to be support cells that only maintain homeostasis and support neuronal function. More recently, astrocytes have been shown to both express cytokine receptors and PRRs, and to secrete cytokines and chemokines (Abbott et al., 2006; Dong and Benveniste, 2001). During homeostatic and/or immune perturbations, astrocytes undergo reactive astrogliosis (which involves morphological changes, hypertrophy, proliferation and altered gene expression) (Pekny and Pekna, 2016) and can take part in both pro- and anti-inflammatory responses (Sofroniew, 2009). In addition, reactive astrocytes form scar tissue, typically surrounding lesions and abscesses, helping to limit the spread of infection and/or excessive inflammation (Sofroniew, 2009).

Neurons, oligodendrocytes, endothelial cells and ependymal cells (ciliated glial cells that line ventricular walls in the brain) have varying degrees of immune reactivity and participate in neuroimmune responses to infection, but their roles in antifungal immunity are less well defined. As such, we do not discuss them in detail here. For more information on the role of these cells in antifungal immunity, we refer readers to a recent review (Reyes and Shinohara, 2022).

### The extracellular matrix

In addition to its diverse cell populations, the CNS contains an ECM, an acellular, proteinaceous scaffold that not only provides structural support but also participates in physiological processes such as cell migration, immunity, and solute transport (Ghorbani and Yong, 2021). The ECM accounts for 10–20% of the total volume of the adult human brain (Holm Nielsen et al., 2025) and consists of basement membranes, perineuronal nets and the interstitial matrix (Tewari et al., 2022). Basement membranes, composed of collagen, laminins and heparan sulphate proteoglycans, line the parenchymal side of cerebral microvessels and thus reinforce the integrity of CNS barriers (Jang et al., 2020; Bignami et al., 1993). The interstitial matrix is dispersed among cells and contains hyaluronan, tenascins and chondroitin sulphate proteoglycans and has a high hydration capacity that helps to maintain extracellular space volume (Jang et al., 2020). Perineuronal nets are composed of hyaluronan, lecticans, tenascin-R and linking proteins (Miao et al., 2014), and they surround neuronal cell bodies and stabilise synapses. Although the ECM itself is not directly antimicrobial, it supports the structural integrity of the BBB and BCSFB, thereby limiting pathogen entry into the CNS parenchyma. Certain ECM components can also serve as adhesion sites for pathogens (Tomlin and Piccinini, 2018). Moreover, the ECM binds cytokines and chemokines, enhancing the recruitment of immune cells to sites of injury or infection (Jang et al., 2020; Sorokin, 2010). Finally, ECM components also actively participate in immune processes by modulating or amplifying immune responses (Jang et al., 2020; Sorokin, 2010).

In summary, CNS immunity against fungal pathogens relies on a multilayered defence system comprising physical barriers (meninges, BBB, BCSFB, ECM), immune-active border regions, and specialised resident and recruited immune cells. These components work in concert to detect, contain and clear fungal invaders while preserving neural integrity. However, despite these robust defences, certain fungal pathogens have evolved sophisticated strategies to breach CNS barriers. By exploiting structural vulnerabilities, modulating host immune responses or directly damaging barrier components, these pathogens can gain access to the parenchyma. Understanding these entry mechanisms is critical for elucidating the pathogenesis of CNS mycoses and developing targeted interventions, as discussed in the next section.

## Mechanisms of fungal entry into the brain

As CNS fungal infections typically originate in peripheral infection sites, penetration of the CNS barrier systems is an important step in fungal invasion of the CNS. Pathogenic fungi infiltrate the brain via three primary mechanisms: (1) the ‘Trojan horse’ mechanism, in which fungal cells hijack and hide within host immune cells that are recruited into the brain (Fig. 2A) (Scherer et al., 2020); (2) paracellular transport, in which fungal cells disrupt endothelial tight junctions or compromise barrier integrity (Fig. 2B) (Cain et al., 2019); and (3) transcytosis, which is an active transport process that enables fungi to move through endothelial cells without disrupting the integrity of the BBB (Fig. 2C) (Strickland and Shi, 2021). Fungi often employ a combination of these mechanisms to infiltrate the CNS (Strickland and Shi, 2021; Santiago-Tirado et al., 2017), which highlights the virulence and versatility of neurotropic fungi in overcoming the protective barriers of the CNS, enabling their dissemination and the development of often life-threatening infections.

### The Trojan horse mechanism

The Trojan horse mechanism is mostly used by neurotropic fungi such as *Cryptococcus neoformans* and *Candida albicans*, which exploit different immune cells to breach the BBB and invade the CNS. *C. neoformans* hijacks infected monocytes and macrophages to traverse capillaries and the microvasculature of the BBB (Heung and Hohl, 2019; Fu and Drummond, 2020). *In vitro* imaging studies utilising a BBB model composed of immortalised human cerebral microvascular endothelial cells co-cultured with primary human monocyte-derived macrophages showed that *C. neoformans*-containing phagocytes breached the BBB via transendothelial pores and infiltrated the CNS within 6 h post-infection (Santiago-Tirado et al., 2017; Heung and Hohl, 2019).

The dual role of monocytes during *C. neoformans* infection is striking: although they release inflammatory cytokines [e.g. interferon-gamma (IFN-γ), interleukin (IL)-2, IL-12] to promote fungal clearance and adaptive immunity (Pitzurra et al., 2000; Lafont et al., 2024), they also paradoxically aid in the dissemination of the fungus to other organs (Fu and Drummond, 2020). For instance, in the periphery, C-C chemokine receptor type 2 (CCR2)^+^ lymphocyte antigen 6 (Ly6C)^hi^ inflammatory monocytes (a subset of inflammatory monocytes that play a role in early immune responses to fungi and can control fungal growth via oxidative bursts) infiltrate the lungs to aid in the containment of infection; however, if their fungicidal capacity is overwhelmed, they end up transporting the fungus to lymph nodes and other tissues, including the CNS (Heung and Hohl, 2019). *In vivo* murine studies by Charlier et al. (2009) support this paradox, finding that *C. neoformans*-infected monocytes significantly enhance fungal dissemination to the spleen, lungs and brain compared to free yeasts. Additionally, they showed that the pharmacological depletion of monocytes using clodronate or their impaired recruitment significantly reduced the fungal burden in various tissues, highlighting how monocytes serve as important vectors for fungal dissemination (Charlier et al., 2009). These findings directly mirror HIV-associated cryptococcosis in humans, whereby impaired monocyte function increases CNS dissemination risk, explaining the high prevalence of fatal cryptococcal meningitis in immunocompromised patients (Monari et al., 1997).

Similarly, *C. albicans*, a leading cause of fungemia (the presence of fungi or yeast in the bloodstream), employs a comparable Trojan horse strategy to cross the BBB, particularly in immunosuppressed individuals (Scherer et al., 2020; Xiao et al., 2022). *In vivo* studies using zebrafish larvae have revealed that *C. albicans* uses host macrophages and neutrophils to cross endothelial barriers (Scherer et al., 2020). Zebrafish larvae are especially useful for modelling human CNS fungal infections owing to their transparent bodies and fully functional innate immune systems, which enable real-time visualisation of host–pathogen interactions. Moreover, the structural and functional features of the BBB, including tight junction proteins such as claudin-5 and zonula occludens-1, as well as innate immune responses, are highly conserved between zebrafish and humans (Fleming et al., 2013; Gomes and Mostowy, 2020), making this model highly relevant for studying fungal CNS invasion. These interactions between fungi and host immune cells highlight the intricate balance between host immunity and fungal pathogenesis, where the very cells tasked with protecting the CNS are co-opted to facilitate its invasion.

### Paracellular transport

Paracellular transport is a less frequently used invasion mechanism, and is employed by *C. albicans*, *Aspergillus fumigatus* and *C. neoformans*. *C. albicans* uses a combination of Trojan horse (Scherer et al., 2020) and paracellular transport (Ibrahim et al., 1995) mechanisms for BBB traversal. Studies have demonstrated that *C. albicans* secretes virulent hydrolytic enzymes, including phospholipase B, which degrades proteins (such as claudins and occludins) that form the tight junctions between endothelial cells and are essential for maintaining the integrity of the BBB (Ibrahim et al., 1995). This enzymatic activity thus weakens the endothelial barrier, facilitating paracellular traversal and fungal dissemination into the brain parenchyma. Moreover, under host conditions, *C. albicans* forms invasive pseudohyphae (elongated chains of yeast cells that resemble true hyphae but lack complete septation), which exert mechanical forces on endothelial layers, exacerbating damage to tight junctions and further increasing barrier permeability (Lossinsky et al., 2006).

Similarly, the filamentous opportunistic fungus *A. fumigatus* employs a dual mechanism of paracellular transport to compromise BBB integrity (Snarr et al., 2020). In addition to using hyphae to apply direct physical pressure to weaken the endothelial barrier, *A. fumigatus* also secretes virulence factors such as elastinolytic aspartic proteinase, which hydrolyses key basement membrane proteins, including collagen and laminin (Curty et al., 2014). The degradation of these structural proteins destabilises the basement membrane, removing critical support for endothelial cells. Additionally, it renders tight junctions more vulnerable to the mechanical stress exerted by fungal hyphae, which promote paracellular transport by forcing apart adjacent endothelial cells as they extend, thereby creating gaps that facilitate fungal traversal across the barrier (Snarr et al., 2020).

This virulence strategy is also employed by *C. neoformans*, which secretes urease that catalyses urea to ammonia (Shi et al., 2010). Ammonia is highly toxic to endothelial cells and disrupts BBB integrity by alkalising the local microenvironment, which triggers oxidative stress and cytoskeletal remodelling, leading to the breakdown of tight junction proteins and increased paracellular permeability, thereby promoting fungal migration into the brain (Shi et al., 2010; Tseng et al., 2015). Studies using urease-deficient *C. neoformans* strains in both *in vitro* BBB models (human brain microvascular endothelial cells) (Shi et al., 2010) and *in vivo* murine infection models (Olszewski et al., 2004) have demonstrated that these strains have significantly reduced CNS invasion compared to wild-type strains. These findings are relevant to human disease, as urease expression was shown to promote microvascular sequestration in brain tissue, a pathological hallmark of fatal cryptococcal meningoencephalitis in immunocompromised patients (Olszewski et al., 2004).

### Transcytosis

Fungi can also traverse the BBB through transcytosis, an active transport process that allows them to cross intact endothelial cells by utilising host transport pathways. Unlike paracellular traversal, which disrupts intercellular junctions and compromises barrier integrity, transcytosis enables fungal passage without causing significant damage to the endothelial layer. For example, *Rhizopus oryzae* enters the brain through receptor-ligand mediated transcytosis (Liu et al., 2010). *R. oryzae* is the primary causative agent of mucormycosis, a rare but aggressive angioinvasive fungal infection that is frequently seen in immunosuppressed individuals (Bhattacharyya et al., 2024) and in patients with diabetic ketoacidosis (Gayathri and Soundarya, 2024). In regions in which the rate of uncontrolled diabetes is high, such as India, for example, the prevalence of mucormycosis has been reported at 14 cases per 100,000 people, which is 80 times higher than that in higher-income countries (Bhattacharyya et al., 2024). In these patients, hyperglycaemia and acidosis increase host iron availability and impair immune function, creating conditions that facilitate fungal invasion (Baldin and Ibrahim, 2017; Gayathri and Soundarya, 2024).

*In vitro* studies using human umbilical vein endothelial cells (HUVECs) have demonstrated that receptor–ligand-mediated transcytosis of *R. oryzae* is driven by its interaction with the endothelial receptor glucose-regulated protein 78 (GRP78; also known as HSPA5), which facilitates fungal endocytosis and host cell invasion (Liu et al., 2011). Liu et al. (2010) found that silencing GRP78 expression in HUVECs reduced fungal invasion by over 60% compared to that in cells treated with a non-targeting siRNA control. This mechanism was further validated in murine models of diabetic ketoacidosis, in which administration of anti-GRP78 antibodies prior to infection resulted in a 65% reduction in brain fungal burden and significantly attenuated endothelial damage relative to that in untreated controls (Liu et al., 2010). These cellular and animal models closely mimic conditions observed in human mucormycosis, particularly in diabetic patients, in whom elevated glucose and iron levels upregulate GRP78 expression, enhancing fungal adhesion and penetration (Al-Huthaifi et al., 2024; Reyes and Shinohara, 2022).

Likewise, *C. albicans* utilises transcytosis to enter the CNS via invasins, which are specialised fungal surface proteins that bind host receptors and trigger fungal uptake (Al-Huthaifi et al., 2024; Reyes and Shinohara, 2022). Two key invasins, agglutinin-like sequence 3 (Als3) and heat shock protein 70-related Ssa1, mediate binding to the brain endothelial receptor glycoprotein 96 (gp96), facilitating fungal internalisation across the BBB (Jong et al., 2012). *In vitro* studies using human brain microvascular endothelial cells have demonstrated that blocking gp96 or disrupting the expression of Als3 or Ssa1 led to a 50-70% reduction in fungal invasion and host cell damage, compared to that in untreated or wild-type controls (Liu et al., 2011). These cellular models are relevant to human disease because Als3 and Ssa1 are expressed during human *C. albicans* infections, and gp96 (also known as HSP90B1 in human) is present on human endothelial cells, including those at the BBB (Liu et al., 2011). This highlights how *C. albicans* not only exploits existing receptor-mediated transport systems but may also fine-tune the expression of its adhesins and invasins to ensure efficient and targeted invasion of the CNS.

Another well-documented example of transcytosis is seen in *C. neoformans*, which uses hyaluronic acid from its capsule (a protective polysaccharide-rich outer layer that enables immune evasion and shields fungal cells from host immune defences), to bind CD44 receptors on human brain endothelial cells (Al-Huthaifi et al., 2024). Simultaneously, *C. neoformans* exploits human host-derived inositol, a monosaccharide abundantly present in the brain, which is important for neuronal signalling (Wang et al., 2021), to activate its own fungal inositol transporter, further enhancing CD44-mediated endocytosis and CNS entry (Reyes and Shinohara, 2022; Al-Huthaifi et al., 2024). The relevance of this mechanism has been further demonstrated in *in vivo* studies, in which CD44-deficient mice infected with *C. neoformans* showed significantly prolonged survival, lower fungal burden in the brain and CSF, and fewer brain lesions compared to wild-type controls (Jong et al., 2012). Although mouse models cannot fully recapitulate human disease, the conservation of CD44 expression and inositol abundance across species suggests that this invasion mechanism is relevant to human cryptococcal meningoencephalitis.

### Other routes of entry into the brain

Although haematogenous dissemination is the primary route for many fungal pathogens, fungi can also invade the CNS through alternative pathways. One such route is direct neural invasion, whereby peripheral and cranial nerves, such as the olfactory nerve, serve as direct pathways into the brain. This has been documented in *R. oryzae*, which can enter the CNS directly via the nasal cavity and olfactory bulbs, leading to meningitis and cranial nerve damage (Wang et al., 2022b). This direct neural invasion is particularly dangerous as it allows fungi to directly access the CNS without encountering systemic immune defences.

Beyond neural pathways, fungi can also exploit regions of the brain in which the BBB is naturally absent or structurally weakened. These include not only the BCSFB at the choroid plexus but also areas like the median eminence and other circumventricular organs, where there is direct blood–CNS communication (Thompson et al., 2022). The choroid plexus, in particular, is susceptible owing to its fenestrated capillaries and limited resident immunity (Thompson et al., 2022). For instance, cases of cryptococcal choroid plexitis, caused by *C. neoformans*, have been documented even in immunocompetent individuals, presenting with radiological signs such as dilated perivascular spaces, hydrocephalus and choroid plexus masses (Kumari et al., 2010).

CNS fungal infections may also occur due to breaches of protective barriers from trauma or medical procedures. Traumatic injuries, such as open head wounds, skull fractures or penetrating injuries, provide direct exposure of neural tissues to environmental fungal spores (Carod-Artal, 2018). This is particularly notable in trauma cases involving mucormycosis, and while mucormycosis typically affects immunocompromised individuals, fatal CNS involvement has also been reported in immunocompetent trauma patients, with overall mortality for CNS mucormycosis exceeding 80% in clinical series (Arfaj et al., 2021; Berczy et al., 2017).

Neurosurgical procedures such as shunt placements or craniotomies also represent significant iatrogenic entry routes (Lahoti and Berger, 2013). Fungi can be introduced via contaminated instruments or implants, or as postoperative complications (Murthy, 2007). One review highlights *Candida* ssp. as a common culprit of CNS infection via shunts and ventricular drains, particularly in infants and immunocompromised hosts (Bhalla et al., 2018). Additionally, nosocomial outbreaks of *Cryptococcus*, *Candida* and *Aspergillus* have been linked to neurosurgical settings, with up to 10% incidence in some post-transplant and post-operative patient cohorts (Wilson, 2015). For example, one study involving 74 patients with CNS fungal infections in Egypt, in which 85% were immunosuppressed, reported *Cryptococcus* (53%), *Candida* (19%) and *Aspergillus* (15%) as the leading pathogens of infection (Khallaf and Kasim, 2019). In these populations, neurosurgical complications such as hydrocephalus often necessitated CSF shunting, further increasing the risk of fungal entry, emphasising the importance of strict aseptic techniques and early antifungal prophylaxis in high-risk patients.

CNS fungal infections arise from the capacity of pathogenic fungi to breach the brain's physical barriers through diverse and often synergistic mechanisms. Whether by hijacking immune cells in Trojan horse strategies, dismantling tight junctions to enable paracellular passage or exploiting receptor-mediated endocytosis for transcytosis, fungi have evolved highly adapted methods for traversing the BBB and related CNS interfaces. These mechanisms are further compounded by alternative entry routes, including direct neural invasion, regions of natural barrier vulnerability and iatrogenic trauma, especially in immunocompromised or critically ill patients. The complexity and redundancy of these entry strategies highlight the complexity of fungal neuropathogenesis and the need for proactive, mechanism-informed approaches for prevention and treatment.

## Mechanisms underlying neuroinflammation and CNS damage in fungal infections

CNS invasion by fungi provokes neuroinflammation through convergent detection of pathogen and host-derived molecules recognised by specialised surface and intracellular receptors expressed by neuroglial cells. In this section, we will discuss how resident glia transduce these cues to drive the production of cytokines, chemokines and reactive oxygen species (ROS) to coordinate local antifungal responses and recruitment of peripheral leukocytes. In addition, we examine how fungal invasion-induced tissue damage generates diverse damage-associated immunogens that can shift the inflammatory balance toward either effective pathogen control or harmful collateral injury. Fig. 2 provides a summary of neuroimmune events that occur during CNS infections by fungi.

### Neuroinflammation arising from fungal recognition by resident immune cells

Antifungal immune responses are initiated when receptors on the surface of resident brain cells interact with fungal antigens known as pathogen-associated molecular patterns (PAMPs; Box 1) (Reyes and Shinohara, 2022; Ramirez et al., 2023), or with host damage-associated molecular patterns (DAMPs; Box 1) that are produced during CNS fungal invasion and growth (Kono and Rock, 2008). PAMPs are evolutionarily conserved molecular motifs that are unique to pathogens (such as bacteria, viruses, fungi and parasites) and are recognised by PRRs on the host's innate immune cells, causing a cascade of inflammatory intracellular signalling (Fig. 1) (Mogensen, 2009). Most common among fungal PAMPs are cell wall polysaccharides and proteins, and fungal nucleic acids (Box 1, Table 2).

Box 1. Dual alarm systems in fungal neuroinvasion: pathogen-associated molecular patterns and damage-associated molecular patterns During fungal neuroinvasion, immune activation is triggered by two alarm systems: pathogen-associated molecular patterns (PAMPs) on the fungal cells and host-derived damage-associated molecular patterns (DAMPs) released during injury.

**PAMPs**

Fungal PAMPs are evolutionarily conserved structural and/or secreted molecular motifs that are found in pathogenic fungi but are absent from mammalian cells. Host immune cells recognise them as ‘non-self’ using a system of germline-encoded receptors known as pathogen recognition receptors (PRRs). PRR activation triggers downstream events aimed at destroying the invading fungal pathogens, including phagocytosis, cytokine release and the activation of antimicrobial effector pathways. Fungal PAMPs encompass cell wall components (e.g. α- and β-glucans, mannans, chitin/chitosan), secreted proteins, and a range of microbial molecules, including lipids, polysaccharides and nucleic acids (Brown et al., 2024). For example, *Candida albicans* PAMPs include N- and O-linked mannoproteins (Netea et al., 2006), chitin (Brown et al., 2024) and candidalysin, a secreted peptide that is sensed by microglia and has been shown to drive neutrophil influx (Drummond et al., 2019). *Aspergillus* spp. have glucans, chitins and galactomannan that are recognised as PAMPs (Shankar et al., 2024), and fungal nucleic acids released during lysis may also function as PAMPs, detected by intracellular PRRs that recognise pathogenic genetic material (Biondo et al., 2011).

Some fungal pathogens evade central nervous system immunity by masking cell surface PAMPs. For example, *Histoplasma capsulatum* masks β-1,3-glucans, mannoproteins and the Toll-like receptor (TLR)2-active adhesin Yps3p, with non-immunogenic α-1,3-glucans in its yeast phase (Rappleye et al., 2007). *Cryptococcus neoformans* employs a glucuronoxylomannan/galactoxylomannan capsule to shield its α-glucan, β-glucan, chitin, chitosan and melanin, thereby reducing phagocytosis (Al-Huthaifi et al., 2024).

**DAMPs**

DAMPs are endogenous molecules released from stressed, injured or dying host cells that activate innate recognition receptors. During fungal neuroinvasion, several DAMPs have been implicated in neuroinflammation, and these include (1) the extracellular matrix (ECM) fragments (biglycan and decorin) generated by fungal enzyme digestion of the ECM, which activate TLR2/4 (Babelova et al., 2009; Ghorbani and Yong, 2021); (2) adenosine triphosphate and high-mobility group box 1 released from dead or dying cells, which activate TLR4 on astrocytes (Minkiewicz et al., 2013) and microglia (Irzan et al., 2025); (3) S100 calcium-binding protein B released from damaged astrocytes and microglia, which activates microglia (Adami et al., 2001); and (4) host-derived double-stranded DNA released during death, which activates interferon-inducible protein AIM2 or cyclic GMP-AMP synthase–stimulator of interferon genes receptors on microglia, astrocytes and neurons (Reyes and Shinohara, 2022).

The interplay between PAMP recognition of fungi and DAMP amplification ultimately determines the outcome of antifungal immunity, determining whether host defence is effective or whether it escalates into destructive immunopathology.

**Table 2.** Pattern recognition receptor signalling in resident brain cells during fungal CNS infection

Cell type	Fungus/PAMP/DAMP	Model(s)	PRR	Signalling molecules	Effector molecules	Cellular outcome	References
Microglia	β-glucans (*S. cerevisiae*)	Ms (primary, IV)	Dectin-1	SFKs, Syk	ROS	Phagocytosis of fungal β-glucans	Shah et al., 2008; Hadas et al., 2010
	Zymosan (*S. cerevisiae*)	Ms (primary, IV)	TLR2	NF-κB	IL-1β, IL-2, IL-6, IL-10, TNF-α	Cytokine production	Shah et al., 2008
	Zymosan (*S. cerevisiae*)	Ms (primary)	CR3	−	−	Opsonised/unopsonised phagocytosis	Hadas et al., 2010
	Yps3p (*H. capsulatum*)	Ms (primary)	TLR2	MyD88, IRAK2, TRAF6, NF-κB	CCL2	Microglial activation	Aravalli et al., 2008
	*C. neoformans* (whole yeast, opsonised)	Ms (*in vivo*), Hm (primary)	TLR2, CD14, complement receptors	MyD88, tyrosine phosphorylation	IL-6, MIP-1α/β, RANTES	Phagocytosis, T-cell recruitment	Shimoda et al., 2006; Redlich et al., 2013; Reguera-Gomez et al., 2024; Goldman et al., 2001; Lee et al., 1995
	*C. neoformans* (avirulent, non-melanogenic)	Ms (*in vivo*)	−	−	IL-12, TNF-α, IL-1β	Effective antifungal response	Barluzzi et al., 2000
	*C. neoformans* (whole yeast)	Ms (*in vivo*)	−	−	CXCL-10, IFN-γ	Increased microglial activation, recruitment of CD4^+^ T cells to the brain	Aguirre and Miller, 2002; Eschke et al., 2015
	*C. albicans* (yeast, candidalysin)	Hm (IV), Ms (IV, *in vivo*)	TLR2, dectin-1/2/3, Mincle, CD11b, TLR4	Syk–CARD9– BCL10–MALT1, NF-κB, MAPK, c-Fos	IL-1β, IL-6, TNF-α, CXCL1, MMP-9, NO, IFN-β	Phagocytosis, inflammasome activation, antifungal clearance, neutrophil recruitment	Gandhi et al., 2020; Orsi et al., 2014; Maneu et al., 2011; Drummond et al., 2019; Flores-Maldonado et al., 2023
	Aβ-like peptides produced by *C. albicans*	Ms (*in vivo*, IV)	TLR4	−	−	Enhanced fungal clearance	Wu et al., 2023
	*A. flavus* clinical isolates	Hm (IV)	TLR1/2/5/7/9	−	IL-1β, IL-6, IL-8, IL-10, IL-17, MMP-9		Gandhi et al., 2020
	*A. flavus* conidia	Ms (*in vivo*)	−	−	IL-12p70, IL-12/IL-23 p40 subunit, IL-6, IFN-γ	Activated microglia, neuronal necrosis and pyknosis	Anand et al., 2015
	*A. fumigatus* hyphae and conidia	Hm (IV)	Complement receptors	−	−	Increased microglial phagocytosis	Rambach et al., 2005
Microglial DAMPs	ATP	Various (Hm, Ms, IV)	P2RX7	−	IL-1β, ROS	Neurotoxic overactivation	Rodrigues et al., 2015
	HMGB1	Various (Hm, Ms, IV)	TLR4	−	−	Increased neuroinflammation	Irzan et al., 2025
	S100β	Rn (primary), Ms (IV)	−	−	NO	Microglial activation	Adami et al., 2001
	dsDNA (host cell)	Various (Hm, Ms, IV)	TLR9, cytosolic PRRs	−	IFNs, CCL3/5, CXCL2, TNF-α, IL-6	Increased neuroinflammation	Suptela and Marriott, 2023
	Biglycan	Ms (*in vivo*, IV)	TLR4	NF-κB	TNF-α, IL-1β, IL-6	Increased neuroinflammation	Xie et al., 2020
Astrocytes	*C. gattii*/*C. neoformans*	Hm (astrocytoma, primary), Ms (*in vivo*)	HLA-DR (MHC-II)	−	IL-6, NO	Fungal uptake, limit GXM accumulation, fungal killing	Olave et al., 2017; Reguera-Gomez et al., 2024; Lee et al., 1994
Astrocytic DAMPs	ATP	Hm (primary)	P2RX7	Pannexin-1, NLRP2–ASC–pro-caspase-1	IL-1β, IL-18	Glutamate/GABA release→neurotoxicity	Minkiewicz et al., 2013
	dsDNA (host cell)	Various (Hm, Ms, IV)	TLR9, cytosolic PRRs	−	IFNs, CCL3/5, CXCL2, TNF-α, IL-6	Neuroinflammation	Suptela and Marriott, 2023

Aβ-like peptide, amyloid beta-like peptide; ASC, apoptosis-associated Speck-like protein containing a CARD; ATP, adenosine triphosphate; BCL10, B-cell lymphoma/leukaemia 10; CARD9, caspase recruitment domain-containing protein 9; CCL, C-C motif chemokine ligand; CD, cluster of differentiation; c-Fos, cellular proto-oncogene Fos (AP-1 transcription factor component); CXCL, C-X-C motif chemokine ligand; CR3, complement receptor 3; DAMP, damage-associated molecular pattern; GABA, gamma-aminobutyric acid; GXM, glucuronoxylomannan; HLA-DR, human leukocyte antigen – DR isotype; Hm, human; HMGB1, high-mobility group box 1; IFN, interferon; IL, interleukin; IRAK2, interleukin-1 receptor-associated kinase 2; IV, *in vitro* cell line; MALT1, mucosa-associated lymphoid tissue lymphoma translocation protein 1; MAPK, mitogen-activated protein kinase; MHC, major histocompatibility complex; MIP, macrophage inflammatory protein; MMP-9, matrix metalloproteinase-9; Ms, mouse; MyD88, myeloid differentiation primary response 88; NF-κB, nuclear factor kappa-B; NLRP2, NLR family pyrin domain-containing 2; NO, nitric oxide; PAMP, pathogen-associated molecular pattern; PRR, pattern recognition receptor; P2RX7, purinergic receptor P2X 7; RANTES, regulated on activation, normal T cell expressed and secreted; Rn, rat; ROS, reactive oxygen species; SFK, Src family kinases; Syk, spleen tyrosine kinase; S100β, S100 calcium-binding protein B; TLR, Toll-like receptor; TNF-α, tumour necrosis factor-alpha; TRAF6, TNF receptor-associated factor 6; Yps3p, yeast phase-specific protein 3.

DAMPs, molecules of host origin that are produced or released as a result of host tissue damage (Box 1), are also recognised by PRRs on the host's innate immune cells, and can activate inflammatory pathways and recruitment of peripheral cells (Fig. 1, Table 2) (Kono and Rock, 2008). DAMPs relevant to CNS fungal infections include host molecules produced owing to the degradation of the ECM, as well as molecules released by dying or dead cells (Box 1). The detection of DAMPs and the subsequent immune activation, when well regulated, aids in tissue repair and serves as a defence against infection in contexts in which PRR recognition has failed to elicit suitable immune responses (Kono and Rock, 2008). In some fungal CNS infection contexts, however, DAMP–PRR signalling contributes to exacerbated inflammation, and dangerously perpetuates fungi-induced inflammation (see ‘Neuroinflammation arising from direct structural damage and injury’ section below).

Resident CNS cells that express PRRs capable of detecting fungal PAMPs and DAMPs include microglia, astrocytes, neurons and oligodendrocytes (for more information on PRR expression in resident immune cells of the CNS, we refer readers to a comprehensive review, Reyes and Shinohara, 2022). Microglia express a broad repertoire of PRRs, including Toll-like receptors (TLR1–13), C-type lectin receptors [e.g. dectin-1, Mincle (also known as CLEC4E), mannose receptor], integrins such as CD11b (also known as ITGAM) and nucleotide-binding oligomerisation domain-containing protein (NOD)-like receptors [e.g. NOD1, NOD2 and NLR family pyrin domain-containing proteins (NLRPs)]. Together, these receptors enable direct detection of fungal PAMPs as well as host-derived DAMPs. This extensive PRR diversity equips microglia with robust fungal-sensing capabilities and positions them as central effectors of innate immunity in the CNS. Upon detection of fungal PAMPs, microglia trigger phagocytosis, produce antifungal ROS, and simultaneously upregulate inflammatory signalling pathways that result in the release of chemokines and cytokines (Koutsouras et al., 2017), which boost fungal clearance by the resident brain cells and recruit peripheral immune cells to the site of infection (Fig. 2) (Drummond et al., 2019).

Astrocytes also express PRRs that detect fungal components and DAMPs (Reyes and Shinohara, 2022; Table 2). They contribute to antifungal defence by releasing cytokines, nitric oxide and other neuroinflammatory mediators (Table 2). Importantly, astrocytes are the major local producers of complement in infected brain tissue, promoting opsonisation of fungal cells and facilitating microglial phagocytosis (Rambach et al., 2008). Table 2 and Fig. 2 highlight the central role of microglia and astrocytes in fungal PAMP and host DAMP recognition during fungal CNS infection.

Neurons and oligodendrocytes primarily propagate neuroinflammatory signals when their PRRs, such as TLRs, and inflammasome pathways are engaged. Rather than mounting robust antifungal responses, their activation amplifies inflammatory cascades that shape the tissue microenvironment (Reyes and Shinohara, 2022). Although their direct involvement in fungal clearance is plausible, this remains to be experimentally confirmed.

In sum, resident immune cells coordinate antifungal defences by coupling local clearance mechanisms with the recruitment of peripheral immune effectors, thereby mounting a multi-layered response to fungal invasion of the CNS (Reyes and Shinohara, 2022; Drummond, 2023).

### The role of peripheral inflammatory cells in fungal CNS infections

Peripheral immune activation normally precedes CNS infection, and that means that a repertoire of primed, pathogen-specific leukocytes (e.g. neutrophils, monocytes, T cells, B cells and natural killer cells) is readily available for recruitment into the CNS to contribute to pathogen clearance. The involvement of these peripheral immune cells in antifungal immunity in the CNS is critical. CD4^+^ cells, for example, release cytokines, such as IFN-γ, that enhance the neuroinflammatory immune response by microglia and recruit monocyte-derived macrophages to contribute to cryptococcal clearance in the CNS (Jarvis et al., 2013; Mohamed et al., 2022). A deficiency in CD4^+^ T cells, as seen in HIV infection/AIDS, greatly increases the risk of cryptococcal meningitis, as illustrated by an analysis of national health data in Botswana that found that, in 2022, the incidence of cryptococcal meningitis was ∼6.6-fold higher in the HIV/AIDS population than in the total population (Milburn et al., 2024).

Recruited neutrophils participate in fungal clearance both directly, by phagocytosing and killing fungal cells, and indirectly, by releasing ROS that compromise fungal cell integrity (Desai and Lionakis, 2018; Rambach et al., 2005). Neutrophil dysfunction, such as in neutropenia (low levels of neutrophils), increases vulnerability to *Candida* infections (Jarvis et al., 2013; Mohamed et al., 2022) and has been shown to be the largest risk factor for chronic disseminated candidiasis (Chen et al., 2025b). Similarly, genetic mutations resulting in caspase recruitment domain-containing protein 9 (CARD9) deficiency have been shown to predispose patients to disseminated candidiasis with CNS involvement (Zhou et al., 2024; Gavino et al., 2014).

Many other types of peripheral immune cells have been shown to respond to fungal infections of the CNS. These include CD8^+^ T cells, along with B cells, granulocytes, monocyte-derived macrophages, natural killer cells, dendritic cells, platelets, mast cells and plasma cells. Table 3 provides a comprehensive summary of the roles of different brain-resident and peripheral immune cells in antifungal immunity of the CNS and highlights the complexity of immune cell responses and interactions in fungal CNS infections.

**Table 3.** Innate and adaptive immune cell contributions to CNS antifungal immunity

Cell type	Key roles in antifungal CNS immunity	Models/contexts	References
Brain-resident cells			
Microglia	Phagocytosis	Ms IV (*Cryptococcus* spp.) Hm/Ms (primary) (*Aspergillus* spp.)	Barluzzi et al., 1998; Rambach et al., 2005
	Cytokine/chemokine production	Ms IV (*Candida* spp.) Hm IV (*Aspergillus* spp., *Candida* spp.)	Drummond et al., 2019; Gandhi et al., 2020
	Complement production	PM (*Aspergillus* spp.)	Rambach et al., 2008
	Formation of fungal-induced glial granulomas	Ms IV (*Candida* spp.)	Wu et al., 2019
	Fungal detection and activation of CD4^+^ T cells	Ms IV (*Cryptococcus* spp.)	Aguirre and Miller, 2002
Astrocytes	Cytokine/chemokine production	Hm (primary) (*Cryptococcus* spp.) Ms *in vivo* (*Candida* spp.)	Woo and Martinez, 2021; Drummond et al., 2019; Reyes and Shinohara, 2022
	NO production	Hm (primary) (Cryptococcus)	Lee et al., 1994; Rambach et al., 2008
	Complement production	PM (*Aspergillus* spp.)	Rambach et al., 2008, Wu et al., 2019
	Formation of fungal-induced glial granulomas	Ms *in vivo* (*Candida* spp.)	Wu et al., 2019; Reyes and Shinohara, 2022
	Dysregulation of brain fluid balance	Ms *in vivo* (*Cryptococcus* spp.) Hm (primary) (*Cryptococcus* spp.) Ms *in vivo* (*Candida* spp.)	Woo and Martinez, 2021; Wu et al., 2019
Endothelial cells	Secretion of inflammatory mediators; BBB regulation; damage during fungal entry	Hm IV (*Cryptococcus* spp.)	Mozaffarian et al., 2000
Neurons	Complement production; potential PRR signalling	PM (*Aspergillus* spp.) Ms *in vivo* (*Candida* spp.)	Rambach et al., 2008; Reyes and Shinohara, 2022
Oligodendrocytes	Complement production; potential PRR signalling	PM (*Aspergillus* spp.) Ms *in vivo* (*Candida* spp.)	Rambach et al., 2008; Reyes and Shinohara, 2022
CNS-infiltrating peripheral cells			
CD4^+^ T cells	IFN-γ and cytokine production; microglia/macrophage activation; contribute to C-IRIS; regulatory subsets limit pathology	Ms *in vivo* (*Cryptococcus* spp.) HIV-ART CS (*Cryptococcus* spp.)	Jarvis et al., 2013; Mohamed et al., 2022; Boulware et al., 2010b
CD8^+^ T cells	Release cytotoxic molecules	HBS (*Candida* spp.) Hm IV (*Candida* spp.)	Breinig et al., 2012
B cells	Antibody production; opsonisation; limit brain dissemination	Ms *in vivo* (*Cryptococcus* spp.)	Dufaud et al., 2018
Granulocytes (neutrophils, eosinophils)	Phagocytosis; oxidative burst; NET formation; trafficking fungi across BBB	Various (Ms IV, Hm IV, Ms *in vivo*, PM, hCSF) (*Aspergillus* spp., *Candida* spp.)	Desai and Lionakis, 2018; Rambach et al., 2008; Scherer et al., 2020
Monocyte-derived macrophages	Antigen presentation; phagocytosis; cytokine/NO/complement production; trafficking fungi across BBB	Various (Ms *in vivo*, ZL, Hm IV, Ms IV, PM) (*Aspergillus* spp., *Cryptococcus* spp., *Candida* spp.)	Scherer et al., 2020; Rambach et al., 2005, 2008
Natural killer cells	Fungal phagocytosis; cytokine release; cytotoxicity	Hm IV (primary) (*Candida spp.*, *Cryptococcus* spp.) Ms *in vivo* (*Cryptococcus* spp.)	Voigt et al., 2014
Dendritic cells	Antigen presentation; initiation of adaptive immunity; phagocytosis	Hm IV (*Cryptococcus* spp.) Ms IV (*Cryptococcus* spp.) Ms *in vivo* (*Candida* spp.) PM (*Aspergillus* spp.)	Rambach et al., 2005
Platelets and mast cells	Recruit eosinophils	Ms *in vivo* (*Cryptococcus* spp.)	De Giovanni et al., 2023
Plasma cells	Limit fungal dissemination	Ms *in vivo* (*Candida* spp.)	Fitzpatrick et al., 2020

BBB, blood–brain barrier; CD, cluster of differentiation; C-IRIS, cryptococcal immune reconstitution inflammatory syndrome; CNS, central nervous system; HBS, human blood samples; hCSF, human CSF; HIV-ART CS, HIV-ART cohort studies; Hm, human; IFN-γ, interferon gamma; IV, *in vitro* cell line; Ms, mouse; NET, neutrophil extracellular trap; NO, nitric oxide; PM, post-mortem; PRR, pattern recognition receptor; ZL, zebrafish larvae.

### Neuroinflammation arising from direct structural damage and injury

Fungal invasion of the parenchyma involves direct or indirect damage to the brain's barrier systems and ECM, often leading to a vicious cycle of neuroinflammation and neuronal injury. Damage to host cells, tissues and ECM can exacerbate inflammatory activation, as brain-resident and peripheral immune cells also express PRRs that detect molecules associated with cell damage (Kono and Rock, 2008). The detection of DAMPs (Box 1) results in a similar upregulation of inflammatory pathways to that initiated by PAMP detection, as this is intended to aid in the clearing of cell debris and prevent further tissue damage.

#### Fungal entry damages CNS barrier systems and the ECM

As outlined above, during CNS invasion by fungi barriers via proteolysis of tight junctions and basement membrane (Ibrahim et al., 1995; Curty et al., 2014), hyphal mechanical forcing (Lossinsky et al., 2006; Snarr et al., 2020) and urease-driven endothelial toxicity (Shi et al., 2010; Tseng et al., 2015; Olszewski et al., 2004), all of which cause damage to BBB cells. Disruption of the BBB exacerbates neuroinflammation, as it allows for the free infiltration of blood-borne substances, peripheral immune cells and plasma proteins, which cause inflammatory activation of microglia and astrocytes (Takata et al., 2021). Additionally, fungal pathogens [such as *Paracoccidioides brasilensis* (Barbosa et al., 2006), *Pneumocystis jirovecii* (carini) (Fox and Smulian, 2001), *C. albicans* (Crowe et al., 2003; Jong et al., 2003) and *C. neoformans* (Stie et al., 2009)] are known to either release ECM-degrading enzymes or to manipulate existing host proteases, such as plasminogen and metalloproteinases, to break down BBB and ECM proteins. Damage to these structures compromises CNS integrity and triggers inflammation; for example, the ECM fragment, biglycan, exacerbates inflammation by acting as a DAMP that activates TLR4, nuclear factor kappa-B (NF-κB) activation and inflammatory cytokine production in murine microglia (Xie et al., 2020). Biglycan has additionally been shown to activate the TLR4–TRIF (also known as TICAM1) signalling pathway in macrophages to promote C-C motif chemokine ligand 5 (CCL5) expression and T-cell infiltration (for comprehensive reviews on the immune effects of these and other ECM components, see Li et al., 2023; Schaefer et al., 2005).

#### Fungal enzymes and secreted factors directly damage host tissues

In addition to morphological changes and replication, fungi secrete enzymes and other factors that can be harmful to host cells. A recent mouse study, for example, used a strain of *C. neoformans* with a *plb1* gene knockout to show that cryptococcal phospholipase B1 enables extensive cortical tissue damage and the formation of large cryptococcomas, plausibly by degrading brain cell membrane phospholipids (Mohamed et al., 2023). This is supported by an older study that used a mouse model to show that clinical strains of *Cryptococcus* with high phospholipase production result in greater CNS pathology, compared to strains with low phospholipase production (Chen et al., 1997). Similarly, secretory factors derived from culture supernatants of patient-derived *Aspergillus* spp. isolates have been shown to induce irreversible cell damage and necrotic cell death in human neurons, astrocytes and microglial cell lines (Speth et al., 2000).

#### Fungal morphological transitions contribute to CNS damage and fungal persistence

Fungal pathogens exhibit dynamic morphological shifts that enhance their ability to invade and persist within the host. Fungi typically grow in three main cellular morphologies: yeast, pseudohyphae and hyphae. The yeast morphology is important for colonisation and rapid dissemination within the host, and for adhesion to host surfaces (Gow et al., 2002). The hyphal and pseudohyphal morphologies (collectively known as filamentous forms) help with tissue invasion, e.g. breaching endothelial barriers during neuroinvasion (Jong et al., 2001), and with host immune cell disruption and lysis to escape phagocytosis (Lo et al., 1997). In a mouse model of neonatal neurocandidiasis, *C. albicans* was shown to transition from yeast to filament forms when traversing the meninges, its filaments causing necrotic brain injury and deeper brain invasion (Flores-Maldonado et al., 2023). Necrotic brain injury further promotes fungal invasion, as necrotic foci are nutrient-rich, immune-poor areas into which the fungal cells can expand (Nathan et al., 2021). Histopathology of human CNS candidiasis likewise reveals mixed yeasts and pseudohyphae (Jurado and López-Panqueva, 2019). *A. fumigatus* similarly shifts from a conidial form into a network of filamentous hyphae that allows it to grow rapidly in blood vessels and penetrate into tissues, resulting in thrombosis, infarction and necrotic lesions (Dagenais and Keller, 2009). Lastly, *C. neoformans* can switch to a giant ‘titan’ morphotype (reaching up to 100 µm in diameter) that cannot be internalised by macrophages (Okagaki et al., 2010) and is highly resistant to phagocytosis, as well as to both chemical and mechanical digestion (Al-Huthaifi et al., 2024).

#### Fungal immune-evasion strategies amplify tissue injury

Fungi also employ various strategies to evade detection and elimination by the host immune system. For example, *Histoplasma capsulatum* yeasts mask β-(1,3)-glucans with a layer of α-(1,3)-glucan to evade phagocytic recognition (Rappleye et al., 2007). Similarly, *A. fumigatus* conidia are coated in dihydroxynaphthalene-melanin, a pigment that helps the fungal cells resist phagocytic killing and oxidative attack (Boucher and Madhani, 2024). *C. neoformans* grows a bulky capsule containing glucuronoxylomannan (GXM), which can induce T-cell apoptosis, and melanin, which impedes phagocytosis by host cells and shields fungal cells against degradation by host enzymes (Al-Huthaifi et al., 2024). *C. neoformans* also sheds the capsular polysaccharide GXM, which induces immunological unresponsiveness (Zaragoza et al., 2009). As such, *C. neoformans* can survive and even replicate within macrophages and microglia (Zaragoza et al., 2009; Mohamed et al., 2022).

#### Host cell death may exacerbate neuroinflammation

**Fig. 3.**
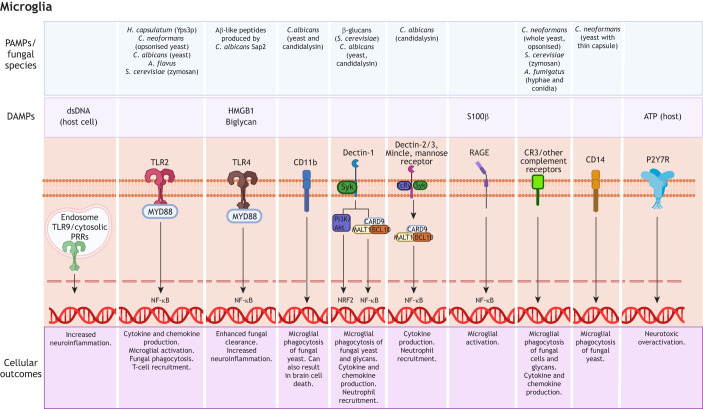
**Microglial PRR recognition of fungal PAMPs and host DAMPs: signalling pathways and effector outcomes.** Schematic summary of PRRs on microglia that detect fungal PAMPs and host-derived DAMPs, with principal adaptor/signalling nodes and dominant outcomes. PAMPs and DAMPs bind to specific cell surface or intracellular PRRs and trigger downstream signalling cascades that lead to microglial activation, cytokine release, phagocytosis and neuroinflammation. Akt, protein kinase B; ATP, adenosine triphosphate; BCL10, B-cell lymphoma/leukaemia 10; CARD9, caspase recruitment domain-containing protein 9; CD, cluster of differentiation; CR3, complement receptor 3; DAMP, danger-associated molecular pattern; dsDNA, double-stranded DNA; HMGB1, high-mobility group box 1; MALT1, mucosa-associated lymphoid tissue lymphoma translocation protein 1; MYD88, myeloid differentiation primary response 88; NF-κB, nuclear factor kappa-B; NRF2, nuclear factor erythroid 2-related factor 2; PAMP, pathogen-associated molecular pattern; PANX1, pannexin-1; PI3K, phosphoinositide-3-kinase; PRR, pattern-recognition receptor; RAGE, receptor for advanced glycation end products; Sap2, secreted aspartyl proteinase 2; Syk, spleen tyrosine kinase; TLR, Toll-like receptor; Yps3p, yeast phase-specific protein 3. Created in BioRender by Dangarembizi, R. (2025). https://BioRender.com/9w4lrg9. This figure was sublicensed under CC-BY 4.0 terms.

Host-initiated programmed cell death is a defence mechanism that aims to restrict pathogens, although fungi can manipulate these cell death programs to evade immune cells and spread to neighbouring cells (Camilli et al., 2021). For example, *A. fumigatus* triggers a calcineurin-dependent, necroptosis-like cell death, which promotes lateral transfer of germinating conidia to neighbouring phagocytic cells (Shah et al., 2016). Activated microglia and recruited peripheral cells produce ROS and reactive nitrogen species that kill fungi but also damage neurons and glia, promoting necroptotic cell death and thus amplifying inflammation (Camilli et al., 2021). Dying host cells release their contents into the extracellular spaces, and some of these components – such as adenosine triphosphate (ATP), high-mobility group box 1 (HMGB1) and double-stranded DNA (dsDNA) (Kono and Rock, 2008) (Box 1) – serve as DAMPs and bind to PRRs on resident brain cells (see Table 2 and Fig. 3) and enhance neuroinflammatory responses.

A classic example of how fungal pathogens induce cell death to subvert antifungal immunity is seen in cryptococcal infections in which GXM, a principal component of the cryptococcal capsule upregulates the Fas ligand in human macrophages, ultimately inducing apoptosis in T cells via caspase-8 and caspase-9 activation (Monari et al., 2003, 2008). *Aspergillus* spp. have been similarly shown to use gliotoxin (a secondary metabolite produced during hyphal growth) to induce apoptosis in human monocytes and dendritic cells (Stanzani et al., 2005).

Collectively, the evidence discussed in this section shows that neuroinflammation in fungal CNS infection can be initiated by tissue injury and PRR sensing of PAMPs, then amplified by DAMPs released from injured or dying cells and the ECM, creating a self-propagating inflammatory loop. Fungal virulence traits such as morphotype switching (hyphae/filaments, titan cells), capsular and melanin shielding, and secreted effectors promote barrier transgression, immune evasion and direct neurotoxicity, thereby fuelling neuroinflammation and further driving neuropathology.

## Pathological implications of neuroinflammation in fungal infections

Neuroinflammation is a central determinant of disease outcome in fungal infections of the CNS. Although inflammatory responses are indispensable for fungal recognition and clearance, they also carry the risk of collateral injury to delicate neural tissue. Both insufficient and excessive immune activation can therefore have pathological consequences, either permitting unchecked fungal growth or causing immune-mediated neurological damage. In this section, we explore the dual-edged role of neuroinflammation in shaping the pathological features of CNS fungal disease.

### Insufficient responses drive fungal persistence and neurological damage

The CNS is an immunologically quiescent environment in which inflammatory responses are tightly regulated, and thus neurotropic fungi exploit this relatively immune privileged nature and multiply. CNS-invading fungal infections are mostly opportunistic in nature, and they disseminate to the CNS in an already compromised host immune environment. Inadequate innate and adaptive immune responses diminish the effective clearance of fungal cells and contribute to rapid fungal multiplication and to severe neurological damage (Lionakis et al., 2023).

Studies that investigated the levels of various cytokines and chemokines in the CSF of individuals with HIV-associated cryptococcal meningitis suggest that well-controlled, but sufficient, neuroinflammation consistently marks clinical benefit. One study in adults diagnosed with HIV-associated cryptococcal meningitis reports that patients who had survived at 2 weeks had ∼5-fold higher CSF IL-6, ∼4-fold higher IFN-γ, >3-fold higher tumour necrosis factor-alpha (TNF-α; also known as TNF) and >2-fold higher IL-8 at baseline than non-survivors, and CSF IFN-γ was positively correlated with fungal clearance (Siddiqui et al., 2005). These results were similar to those observed in another HIV-associated cryptococcal meningitis cohort: at admission, survivors showed higher CSF levels of the inflammatory cytokines IL-8, IL-12p40, IL-17A, IFN-γ and TNF-α, whereas patients who did not survive a 2-week period had higher CSF levels of anti-inflammatory cytokines such as IL-4 and IL-10 (Mora et al., 2015). Again, CSF inflammatory cytokine levels were correlated with lower fungal burden [IFN-γ (*r*=−0.47) and IL-17A (*r*=−0.50)], while IL-4 and IL-10 rose with increasing fungal burden (Mora et al., 2015). Together, these data indicate that insufficient immune activation is a key driver of fungal persistence and CNS damage, underscoring the need for adequate but well-controlled neuroinflammation to achieve fungal control.

### Dysregulated inflammatory responses cause tissue damage

An inflammatory immune response and accompanying phagocytotic activity are essential for clearing infections in the CNS, but excessive or prolonged neuroinflammation causes cell death and tissue damage (Haddow et al., 2010). In a well-regulated, protective neuroimmune response, there are various regulatory processes that assist in limiting and resolving inflammation (Klein and Hunter, 2017); for example, microglia and astrocytes release anti-inflammatory cytokines (e.g. IL-10) to switch off NF-κB/STAT circuits (Lobo-Silva et al., 2016), drive suppressor-of-cytokine-signalling (SOCS) expression (Baker et al., 2009) and polarise glia towards a reparative state, while specialised pro-resolving lipid mediators (resolvins, maresins) reinforce the shutdown and curb further leukocyte infiltration (Valente et al., 2022). Microglia further clear apoptotic cells and cellular debris, facilitating the resolution of neuroinflammation (Chen et al., 2025a). When these mechanisms are suppressed or overwhelmed by fungal persistence and tissue damage, the neuroinflammatory immune response can escalate to fatal levels. Invasive aspergillosis in patients on corticosteroid therapy, for example, is characterised by excessive neuroinflammation and tissue necrosis (Dagenais and Keller, 2009). Corticosteroids greatly impair the ability of phagocytes to kill *A. fumigatus*, while neutrophil recruitment remains intact and results in inflammation-related brain injury that can result in mortality (Dagenais and Keller, 2009).

Perhaps the best-characterised phenomenon of damage due to an excessive immune response to fungal presence is fungal immune reconstitution inflammatory syndrome (IRIS). Fungal IRIS is a pathological condition characterised by excessive immune activation and inflammation that occurs when immune function is restored in individuals with prior immunosuppression and unresolved fungal infections (Delliere et al., 2018). Common triggers of fungal IRIS include antiretroviral therapy initiation in HIV, neutrophil recovery after chemotherapy or stem-cell transplant, modification of immunosuppressive therapy in solid-organ transplant recipients, and postpartum immune changes (Delliere et al., 2018). Fungal IRIS has been reported in infections caused by *Cryptococcus* (Bicanic et al., 2009), *Pneumocystis* (Vaselli et al., 2025), *Aspergillus* (Jung et al., 2015), *Candida* (Berkeley et al., 2008) and *Histoplasma* spp. (Melzani et al., 2019). No common pathophysiological explanation of fungal IRIS exists for all fungi as mechanisms differ depending on the pathogen and the underlying type of immunosuppression, both of which shape the immunological events that lead to IRIS (Delliere et al., 2018). But generally, an inadequate balance between pro-inflammatory [T helper (Th1)/Th17] and anti-inflammatory (Th2/regulatory T cell) response is believed to be the origin of IRIS (for a comprehensive review of this topic, see Delliere et al., 2018).

Cryptococcal IRIS (C-IRIS), which is the most extensively described form of fungal IRIS, is strongly associated with severe T-cell immunosuppression, with large cohort studies reporting average baseline CD4 counts below 50 cells/µl at antiretroviral therapy (ART) initiation (for a summary of cohort and case control data on C-IRIS, refer to Haddow et al., 2010). In C-IRIS patients, initiation of antiretroviral therapy is associated with the restoration and redistribution of Th1 and Th17 cell populations, driving enhanced IFN-γ production in response to pre-existing cryptococcal infection (Delliere et al., 2018). This, in turn, activates innate immune cells – e.g. monocytes, macrophages and neutrophils – and precipitates an exaggerated inflammatory cascade (Delliere et al., 2018). The resulting ‘cytokine storm’ is characterised by elevated levels of pro-inflammatory mediators such as IFN-γ, IL-1β, TNF-α, IL-6, IL-12, IL-18 and IL-23, which strongly amplify peripheral cell recruitment into the CNS and neuroinflammatory signalling (Delliere et al., 2018; Boulware et al., 2010b; Meya et al., 2014). Exaggerated inflammatory activation during cryptococcal IRIS disrupts the BBB, causing cerebral oedema and subsequent CNS injury. In a systematic review of C-IRIS studies, Haddow et al. (2010) reported that 73.7% of C-IRIS cases with available data demonstrated meningeal disease. Reported neurological features associated with C-IRIS include intracranial lesions (cryptococcomas) (Boulware et al., 2010a; Cattelan et al., 2004), raised intracranial pressure (Cinti et al., 2001; York et al., 2005), cranial nerve lesions (Bicanic et al., 2009; Khanna et al., 2006), and motor deficits including hemiparesis and paraparesis (Bicanic et al., 2009; Lawn et al., 2005). In the context of individuals with HIV/AIDS, HIV infection itself can also contribute to neuroinflammation, and this may further contribute to damage in fungal IRIS (Williams and Naudé, 2024; Delliere et al., 2018).

**Fig. 4.**
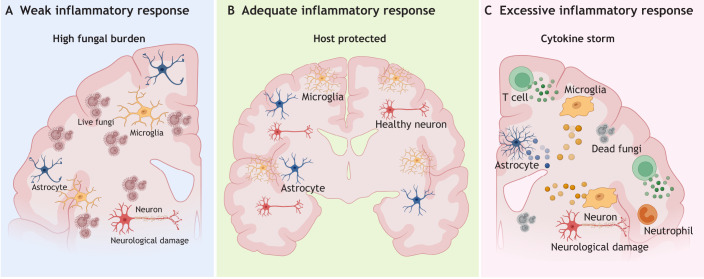
**A schematic representation of the inflammatory balance required for effective antifungal immunity.** (A) Weak neuroinflammatory responses promote fungal proliferation and result in pathogen-related neurological damage. (B) An adequate, well-regulated neuroinflammatory response is protective against fungal infection. (C) Excessive, dysregulated neuroinflammation triggers the excessive production of cytokines resulting in a ‘cytokine storm’, which is maladaptive and causes neurological damage. Created in BioRender. Dangarembizi, R. (2025). https://BioRender.com/v7lm1t6. This figure was sublicensed under CC-BY 4.0 terms.

The evidence reviewed above illustrates that fungal clearance and recovery from fungal brain infections require a finely regulated inflammatory response, and that both a paucity of inflammation and an excess of it can result in tissue damage and poor outcomes for infected individuals. Fig. 4 shows a summary of how the disruption of this delicate balance can drive host damage and neuropathology.

## Summary and future perspectives

Fungal infections of the CNS can be fatal and frequently result in severe neurological damage, yet the mechanisms underlying this neuropathology remain poorly understood. Although neuroinflammation is implicated as a key driver of brain injury during fungal infections, it is overly simplistic to view it as purely detrimental. In this Review, we have highlighted the complexity of the neuroinflammatory response, which involves resident innate immune cells of the CNS that detect pathogens and release pro-inflammatory mediators, as well as peripheral immune cells that mount an adaptive response to clear infection. This coordinated response is critical for fungal clearance; without it, fungi proliferate unchecked in the CNS, resulting in host damage. Conversely, a dysregulated inflammatory response, often accompanied by excessive cytokine release (cytokine storm), can exacerbate neuropathology.

Looking ahead, several important questions remain unresolved. How do neuroimmune and immunometabolic pathways intersect to shape the balance between protective and pathological inflammation? To what extent do fungal-induced disruptions in glymphatic flow and ECM integrity contribute to persistent neuroinflammation and long-term neurological sequelae? An additional, largely unexplored dimension is the potential contribution of the gut–brain axis to neuroinflammation during fungal infection. Gut dysbiosis, which is common in immunocompromised individuals, may influence systemic immune tone, peripheral cytokine release and BBB integrity, thereby shaping the CNS response to fungal pathogens. Future work dissecting how gut-derived microbial or metabolic signals intersect with neuroimmune pathways could uncover novel modulators of inflammation and open new avenues for therapeutic intervention. Addressing these questions will require integrated approaches that combine advanced imaging, single-cell and spatial transcriptomics, and innovative *ex vivo* and *in vivo* models of infection.

From a translational perspective, there is an urgent need to identify biomarkers that predict which patients are most at risk of detrimental inflammatory responses, as well as to define therapeutic windows in which immune-modulating strategies could improve outcomes. Future work should also consider host–pathogen diversity, as regional differences in fungal strains and host immunogenetics may influence disease trajectory. Ultimately, disentangling the dual roles of neuroinflammation, as both a defender against fungal invasion and a driver of neuropathology, will be crucial for informing the next generation of diagnostic and therapeutic interventions for CNS mycoses.
